# Interaction Between Transcription Factor EhPC4 and Polyadenylation Factor EhCFIm25 in *Entamoeba histolytica*: Molecular Characterization and Functional Implications

**DOI:** 10.3390/microorganisms14040809

**Published:** 2026-04-02

**Authors:** Juan David Ospina-Villa, Alondra Cisneros-Sarabia, Rocío Paulina Leal-Acosta, César Augusto Sandino Reyes-López, Absalom Zamorano-Carrillo, Esther Ramírez-Moreno, Laurence A. Marchat

**Affiliations:** 1 Instituto Colombiano de Medicina Tropical, Universidad CES, Medellín 050021, Antioquia, Colombia; jospina@ces.edu.co; 2 Sección de Estudios de Posgrado e Investigación, ENMH, Instituto Politécnico Nacional, Ciudad de México 07320, Mexico; a.cisa9613@gmail.com (A.C.-S.); careyes@ipn.mx (C.A.S.R.-L.); azamorano@ipn.mx (A.Z.-C.); maramirezmo@ipn.mx (E.R.-M.)

**Keywords:** mRNA processing, mRNA synthesis, protein interaction, protozoan parasite

## Abstract

The coordination between transcription and mRNA processing is essential for eukaryotic gene regulation, yet the structural basis of this coupling remains poorly understood in *Entamoeba histolytica*, the protozoan parasite responsible for amoebiasis. In this study, we characterized the interaction between the transcriptional coactivator EhPC4 and the polyadenylation factor EhCFIm25 through an integrated in vitro and in silico approach. Far-Western assays confirmed direct physical interaction between both recombinant proteins. To elucidate the molecular mechanism, we performed 500 ns Molecular Dynamics simulations of full-length EhPC4, identifying high flexibility in its N-terminal region. Protein–protein docking analysis revealed a stable EhPC4-EhCFIm25 complex (Cluster C4) with favorable binding energies (∆G = −11.4 kcal/mol). Notably, heatmap analysis of the interaction interface identified a conserved “hotspot” at the C-terminal end of EhCFIm25 (residues 249–255) that mediates the binding with PC4 without occluding DNA-binding domain (K127 in EhPC4) or RNA-recognition motifs in EhCFIm25. Our findings suggest that EhCFIm25 serves as a molecular scaffold that physically couples transcription and polyadenylation, providing a structural framework for the efficient regulation of virulence-related genes in this parasite.

## 1. Introduction

Pre-mRNA polyadenylation is a fundamental regulatory step in eukaryotic gene expression. This process requires coordinated action of four major multiprotein complexes that recognize specific sequences within the 3′-untranslated region (3′-UTR) of pre-mRNAs. Among these, Cleavage factor I (CFIm), composed of 25 kDa, 59 kDa, and 72 kDa subunits, plays a pivotal role. Notably, CFIm25 binds the UGUA motif, regulates distal poly(A) site selection, recruits additional polyadenylation factors, and is essential for poly(A) tail synthesis in humans [1,2]. We previously reported that CFIm25 is conserved across nearly all human-pathogenic protozoan parasites, underscoring its evolutionary importance [3]. One of the most relevant parasitic diseases is amoebiasis caused by *Entamoeba histolytica* that infects around 50 million people worldwide and is responsible for about 70,000 deaths each year, mainly in developing regions with deficient sanitation and hygiene conditions [4]. The infection begins with ingestion of water and food contaminated with cysts, then trophozoites are released in the intestinal tract where they grow and multiplicate, producing diarrhea and dysentery. They can also cross the intestinal epithelium to invade other organs, mainly the liver, and produce extraintestinal abscesses that can be fatal. Finally, the life cycle is complete when intestinal trophozoites form cysts that are eliminated in the feces [5]. Due to its impact on human health, several molecular mechanisms of *E. histolytica* are investigated, including the mRNA synthesis and processing. Thus, our group previously reported that the EhCFIm25 protein is indispensable for parasite virulence and survival. Silencing of this factor significantly impairs motility and erythrophagocytosis, ultimately inducing cell death. EhCFIm25 recognizes the GUUG motif via a conformational “molecular switch” in the 60–100 region (coil-to-helix transition), a mechanism primarily mediated by residues Lys79, Lys130, and Lys183, alongside the G4 residue of the RNA motif [6,7,8].

Computational protein–protein interaction (PPI) networks have identified EhCFIm25 as a bottleneck protein, bridging the polyadenylation module with broader gene expression regulation networks. Specifically, EhCFIm25 interacts with EhPAP (the central hub of the interactome) and Ehnopp34 [3]. The interaction with EhPAP has been experimentally validated in vitro [9]. Given that PAP is recruited at the onset of transcription in mammals [10,11], the EhCFIm25-EhPAP association suggests that polyadenylation factors may cooperate with the transcriptional machinery from the early stages of mRNA synthesis.

Another key player in this crosstalk is the Positive Coactivator 4 (PC4). This multifunctional nuclear factor facilitates the recruitment of transcriptional activators and general transcription factors, stimulating pre-initiation complex assembly. Beyond transcription, PC4 (and its yeast homolog Sub1) is involved in chromatin condensation, DNA repair, and mRNA processing. Notably, Sub1 modulates termination defects in yeast strains lacking the polyadenylation factor Rna15 (the CstF64 homologue in yeast) [12,13]. In *E. histolytica*, EhPC4 has mainly been studied in the context of transcription. Functional assays revealed that the K127 residue in the FRFPKG motif is required for efficient DNA-binding activity. Additionally, by targeting the EhNUDC gene transcription, PC4 regulates polyploidy and genome stability. Moreover, it also has an essential role in parasite virulence by regulating trophozoite migration and host epithelium destruction [14,15]. To further elucidate the link between transcription and polyadenylation in *E. histolytica*, this study characterizes the interaction between EhPC4 and EhCFIm25 through in vitro and in silico assays.

## 2. Materials and Methods

### 2.1. Expression and Purification of Recombinant EhCFIm25 and EhPC4 Proteins

Competent *E. coli* BL21 (DE3) pLysS bacteria were independently transformed with the pRSET-EhCFIm25 [9] and pRSET-EhPC4 [15] plasmids. The histidine-tagged EhCFIm25 and EhPC4 proteins were expressed in *E. coli* with 1 mM isopropyl β-D-thiogalactopyranoside (IPTG) at 37 °C for 3 h and purified by Ni-NTA affinity chromatography (QIAGEN, Hilden, Germany) under non-denaturing conditions. The identity and integrity of recombinant proteins were confirmed by 10% SDS-PAGE. Both purified proteins were dialyzed against interaction buffer (200 mM NaCl-Tween 5%) for 2 h before being used in further assays.

### 2.2. Far-Western Blotting Assays

Far-Western experiments were performed as we previously described [9]. First, EhCFIm25 was used as bait. EhCFIm25 (40 µg) was subjected to 10% SDS-PAGE and electro transferred to a nitrocellulose membrane that was blocked with 5% non-fat milk in TBST 1X buffer (BSA 2%). Then it was incubated for 4 h at room temperature in binding buffer (NaCl 100 mM, Tris pH 7.6 20 mM, EDTA 0.5 mM, Glycerol 10%, Tween-20 0.1%, Skim milk powder 2%, DTT 1 mM) containing EhPC4 (40 µg/mL) used as prey protein. After washing with TBST 1X, the membrane was incubated with anti-EhPC4 antibodies (1:1000 dilution) for 3 h at room temperature. Finally, goat anti-rabbit IgG (horseradish peroxidase) secondary antibody (Zymed, San Francisco, CA, USA) (1:2000) was added for 3 h at room temperature, and the signal was developed by 3,3′-Diaminobenzidine (DAB) solution (25 mL PBS, 12.5 mg diaminobenzidine Sigma, 2.5 µL H_2_O_2_ 30%). Anti-EhCFIm25 (1:1000) and anti-His (1:6000) antibodies were used as controls.

In another assay, the purified EhPC4 protein (40 µg) was used as bait on the nitrocellulose membrane, and EhCFIm25 was used as prey protein as described above. Proteins were detected using anti-EhCFIm25 (1:1000 dilution) and goat anti-rabbit IgG (horseradish peroxidase) secondary antibody (Zymed) (1:2000). Anti-EhPC4 (1:1000 dilution) and anti-His (1:6000 dilution) antibodies were used as controls.

### 2.3. Three-Dimensional Structure of EhPC4 and Molecular Dynamics Simulation

The 3D structure of the EhPC4 protein (UNIPROT: C4M1H2) was generated using a distance-based protein structure prediction approach by deep learning with the RAPTOR-X server [16]. The stereochemical quality of the selected 3D model of EhPC4 was evaluated on the PROCHECK server [17] and subjected to molecular dynamics simulation (MD) on the GROMACS suite [18] version 5.1, using the OPLS all-atom force-field [19]. Briefly, the EhPC4 structure was solvated using the TIP3P explicit water model in a dodecahedral box with its nearest edge 1.0 nm away from the protein. Chloride ions were added for system neutralization, and all electrostatic interactions were calculated through the Particle Mesh Ewald (PME) approach. Energy minimization was performed using the steepest descent algorithm for 5000 steps. Then, a restrained MD of 1000 ps was performed to allow the solvent to relax; the peptide atoms were harmonically restrained to their position in the model with a force constant of 1000 kJ/mol/nm^2^. All simulations were performed at 300 K and 1 atm pressure. Three replicates of the free MD run were carried out for 500 ns with the same pressure and temperature coupling constants as the constrained experiment. All steps of the simulations were performed using periodic boundary conditions. The stability and conformational changes in the trajectory for EhPC4 structure was characterized by analyzing the root mean square deviation (RMSD), that quantifies how much a structure diverges from another through the trajectory. Therefore, RMSD may indicate the stability of the protein structure during simulation; it may also reflect high flexibility of different regions of the protein structure. The root-mean-square-fluctuation (RMSF) that reveals which regions of the structure are the most mobile and the radius of gyration (Rg), which indicates a measure of a protein compactness, were also analyzed. All these parameters were calculated by tools included in the GROMACS software. Finally, the TTClust program [20] was used to cluster the trajectories and obtain a representative frame for each cluster.

### 2.4. EhPC4-EhCFIm25 Molecular Docking

The representative 3D structures of each cluster obtained from the MD of EhPC4, and the 3D structure of EhCFIm25 protein previously reported by our working group [3] was entered into the LZerD webserver (default parameters were used) [21] to perform protein–protein molecular docking analysis. For each docking assay, the first 10 molecular docking complexes between both proteins were evaluated based on the frequency of contact regions (docking cluster size) and the best multi-score classification of the LZerD server. The ΔG value corresponding to the best docking model of each cluster was estimated using the PRODIGY server [22]. To reveal the amino acids involved in the contacts between EhPC4 and EhCFIm25, LigPlot+ [23] was used. Finally, the Heat Map option in GraphPad Prism v. 8.0.1 was utilized to create the contact map between both proteins.

## 3. Results

### 3.1. Recombinant EhCFIm25 and EhPC4 Proteins Interact with Each Other

**Figure 1:**
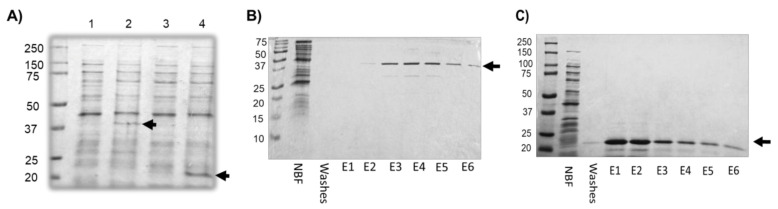
Expression and purification of recombinant EhPC4 and EhCFIm25 proteins. (**A**) Recombinant EhCFIm25 (lane 2) and EhPC4 (lane 4) were expressed in *E. coli* BL21 (DE3) pLysS bacteria with 1 mM IPTG. Lanes 1 and 3, bacterial proteins without the addition of IPTG. Lanes 2 and 4, after adding IPTG. (**B**,**C**) Purification of recombinant EhCFIm25 (**B**) and EhPC4 (**C**) by Ni-NTA affinity chromatography. NBF: Not bound fraction; E1-E6, fractions eluted with 250 mM imidazole. Molecular Weight markers (kDa) are on the left. Black arrows, recombinant proteins.

To gain insights into the relationships between transcription and polyadenylation factors in *E. histolytica*, the interaction between EhCFIm25 and EhPC4 was assessed by Far-Western assays. First, recombinant EhCFIm25 and EhPC4 proteins were expressed in *E. coli* BL21 (DE3) pLysS and appeared as the expected 38 kDa and 21.8 kDa bands, respectively, in gels (Figure 1A). Then, they were purified by NI-NTA affinity chromatography under native conditions as demonstrated by the visualization of a single band at the expected molecular weight (Figure 1B,C).

**Figure 2:**
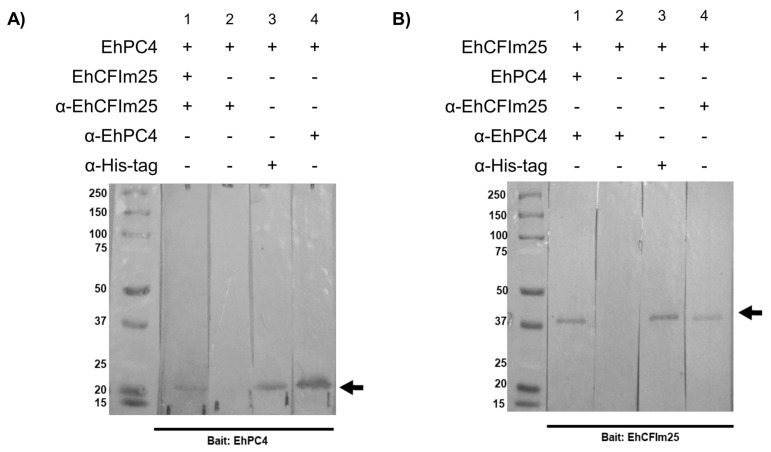
Far-Western assays using recombinant EhCFIm25 and EhPC4 proteins. Purified EhPC4 (**A**) and EhCFIm25 (**B**) were subjected to 10% SDS-PAGE and electro transferred to a nitrocellulose membrane to be used as bait in Far-Western assays. They were incubated with EhCFIm25 and EhPC4 used as prey proteins, respectively, and immunodetected by specific anti-EhCFIm25 and anti-EhPC4 antibodies, respectively. Bait proteins alone were also immunodetected with the corresponding antibody as a control. Molecular Weight markers (kDa) are on the left. Black arrows, bait proteins.

Then, EhPC4 was used as bait on a cellulose membrane (Figure 2A). When EhCFIm25 was used as prey, anti-EhCFIm25 antibodies recognized a 21.8 kDa band that corresponds to the molecular weight of the EhPC4 protein (lane 1), while no signal was obtained in the absence of EhCFIm25 (lane 2). As controls, anti-His and anti-EhPC4 antibodies recognized the same band of 21.8 kDa, which corresponds to the predicted molecular weight for recombinant EhPC4 (lanes 3 and 4). Conversely, a 37 kDa band was detected by anti-EhCFIm25 antibodies when EhCFIm25 was used as bait and EhPC4 as prey (Figure 2B, lane 1), but antibodies did not detect any band in the absence of EhPC4 (lane 2). Controls using anti-His and anti-EhPC4 antibodies recognized the recombinant EhCFIm25 at 37 kDa (lanes 3 and 4). Taken altogether, these results indicate that EhCFIm25 and EhPC4 interact with each other in vitro.

### 3.2. Modeling and Molecular Dynamics Simulation of EhPC4

**Figure 3:**
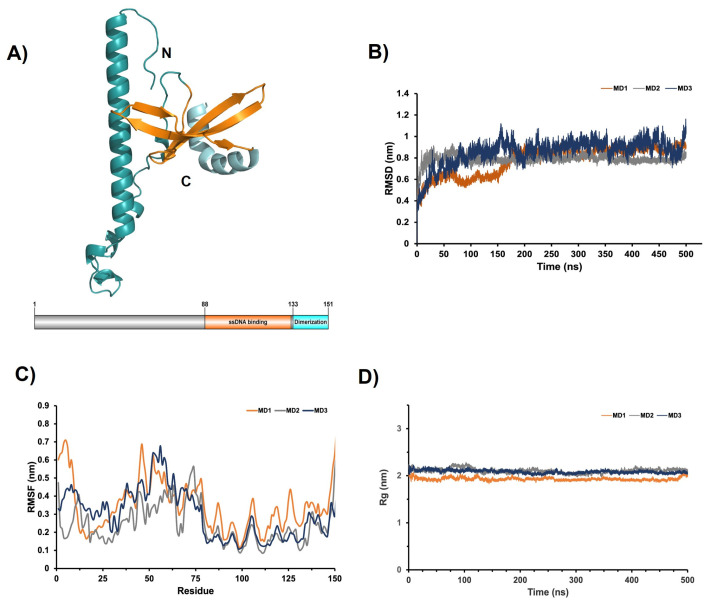
Modeling and molecular dynamics analysis of EhPC4. The full-length EhCF4 (151 residues) was modeled with the RAPTOR X server and submitted to MD for 500 ns using the GROMACS software. MD trajectories were analyzed from three independent replicates (MD1, MD2, MD3). (**A**) Ribbon representation of the three-dimensional structure of full-length EhCF4 modeled with the RAPTOR X server and the molecular organization of the protein is shown in the lower panel. (**B**) Time evolution of α-carbon RMSD, (**C**) RMSF, and (**D**) protein Rg during MD simulation.

Since the crystallographic structure of EhPC4 is not available in the PDB, a full-length 3D model of EhPC4 protein was generated in the RAPTOR-X server. Stereochemical quality analysis showed 93.4% residues in the most favored regions and 6.5% in additional allowed regions according to the Ramachandran plot, suggesting a good quality 3D structure of PC4 for further analysis. The modeled structure of EhPC4 showed a single-stranded DNA (ssDNA)-binding domain, which adopts a conformation consisting of a β-sheet formed by four β-strands (residues 88–132) and a C-terminal dimerization domain (residues 133–151), which is folded into an α-helix (Figure 3A). The ssDNA-binding domain of EhPC4 exhibits a folding similar to the dimeric C-terminal structure of human PC4 (PDB: 1PCF), judging by superimposing the Cα atoms (RMSD 2.5 Å) as well as to the tetrameric DNA-binding domain of human PC4 in complex with a modified nucleic acid (PDB: 6YCS) (RMSD 2.9 Å).

The full 3D model of EhPC4 contains 2.5 times as many amino acids as the crystal structure of the C-terminal domain of its human homolog. Approximately 30% of its residues are structured into an α-helix at the N-terminal end of the protein (residues 11 to 46), which is connected to the C-terminal domains via a large loop (residues 47 to 91). This structural arrangement suggests that the N-terminal helix may adopt different conformations; therefore, MDs were employed to obtain representative structures for docking predictions of the EhPC4-EhCFIm25 complex. During the 500 ns simulation, the RMSD of the Cα atoms for the model remained around 0.8 nm across three independent simulation replicates, indicating no significant differences between the initial and final conformations of EhPC4 (Figure 3B). This structural stability was further supported by analyzing the per-residue RMSF and the Rg from the three MD replicates (Figure 3C,D).

**Figure 4:**
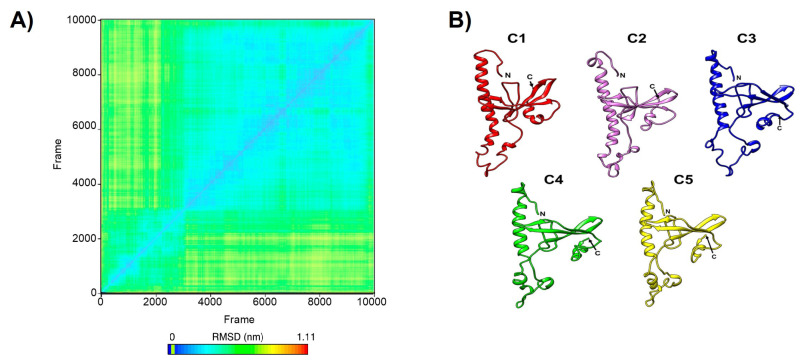
Cluster of EhPC4 (500 ns trajectory). (**A**) 2D plot of RMSD between frames. (**B**) Average representative cluster structures of EhPC4 (500 ns trajectory).

**Figure 5:**
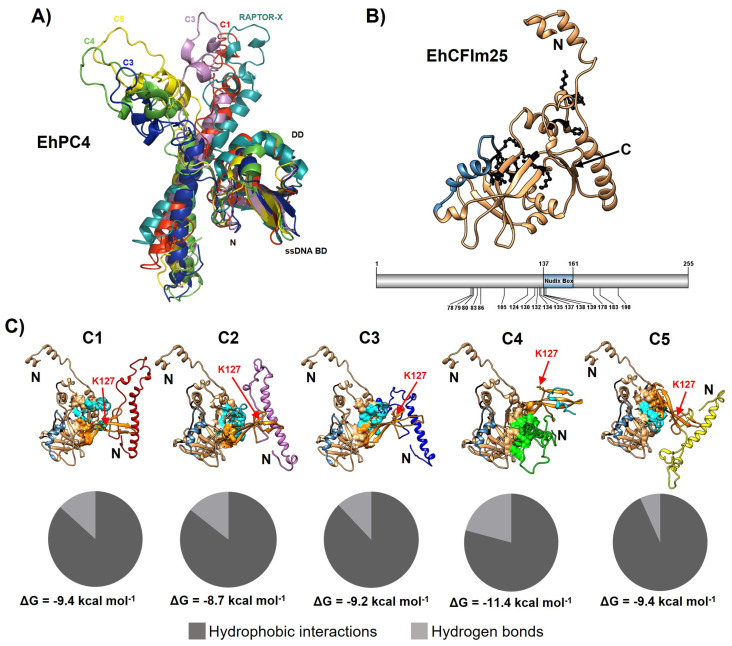
Molecular docking between the five structures obtained from EhPC4 clustering (C1–C5) and EhCFIm25. (**A**) Superposition of the C1-C5 structures from MD cluster analysis and RAPTOR X structure of EhPC4. The ssDNA binding domain (ssDNA BD, residues 88–132) and the dimerization domain (DD, residues 133–151) are shown. (**B**) Structure of the EhCFIm25 protein (255 aa) described in [3] (**upper panel**) and molecular organization of the protein (**lower panel**). The Nudix box (137–161 aa) is in blue and the residues that interact with RNA (GUUG motif) are in black. (**C**) Prediction of the five EhPC4-EhCFIm25 complexes. (**Upper panel**): The interaction interface of the C1–C5 structures of EhPC4 with EhCFIm25 is marked with the surface of each protein. The position of the K127 residue of EhPC4 that binds DNA is indicated with the red arrow. The N-terminal region of each protein is shown with a bold N. (**Lower panel**): The proportion of each kind of interaction and ΔG values are indicated.

Following the concatenation of the MD trajectories from three independent runs, five representative structures were obtained through cluster analysis (Figure 4A,B). The superposition of the five representative EhPC4 models (C1–C5), derived from MD cluster analysis, revealed minor variations within the ssDNA-binding and dimerization domains. The Cα atoms of models C1, C2, and C4 exhibited an RMSD of 2.4 Å relative to the corresponding domains in the initial RAPTOR-X model, while models C3 and C5 showed an RMSD of 2.1 Å and 2.7 Å, respectively. Notably, when the alignment was performed using only the residues from these functional domains, amino acids 39–76 in models C3, C4, and C5 separated from the core of the structure. This movement partially disrupts the N-terminal a-helix of EhPC4 (Figure 5A), with the most pronounced displacement observed in the C4 model. In this model, S54 shifts by up to 52 Å from its position in the initial structure, indicating a significant conformational change in this region following MD.

### 3.3. EhPC4-EhCFIm25 Molecular Docking

**Figure 6:**
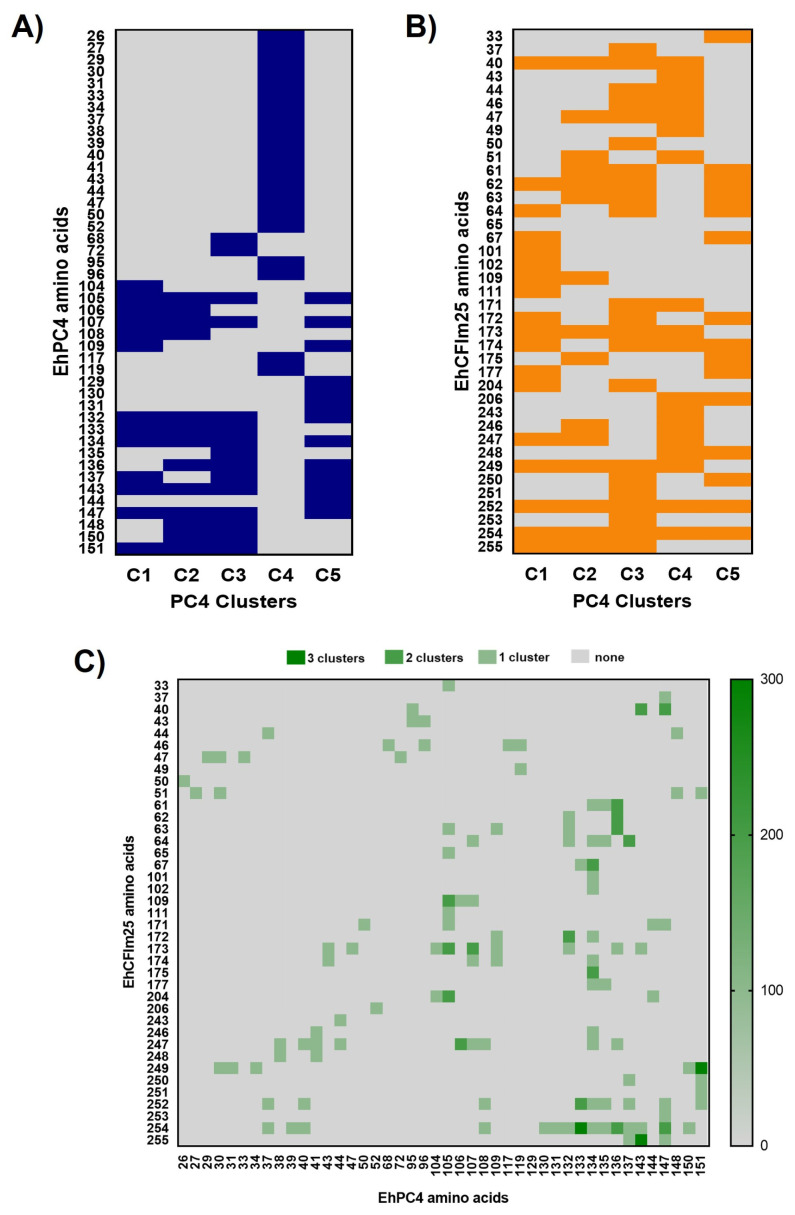
Analysis of the amino acid interactions between EhPC4 and EhCFIm25. (**A**) Amino acids of EhPC4 (C1, C2, C3, C4 and C5) that interact with EhCFIm25. (**B**) Amino acids of EhCFIm25 that interact with EhPC4 (C1, C2, C3, C4 and C5). (**C**) Interactions between EhPC4 and EhCFIm25 that are conserved in 1, 2, or 3 clusters.

The five cluster-derived models of EhPC4 and the previously reported EhCFIm25 3D model (Figure 5B) were used for independent docking predictions. Complex structures were selected based on high LZerD scores and an absence of steric clashes for subsequent analysis and superposition with human PC4 crystallographic structures. The binding energies for the selected docking complexes, estimated with the PRODIGY server, ranged from −8.7 to −11.4 kcal mol^−1^, which falls within the expected range for protein–protein interactions. Docking analysis showed that the complexes between the C1, C2, C3, and C5 cluster models of EhPC4 bind to EhCFIm25 through some amino acids located in the dimerization and ssDNA-binding domains of EhPC4 protein, which could prevent DNA binding. Although the key amino acid K127 remains accessible for DNA binding in these models, the docking complex with the C4 cluster model kept the dimerization and ssDNA-binding domains fully accessible in EhPC4 while bound to EhCFIm25, suggesting that this complex could be biologically relevant (Figure 5C and Figure 6A).

The interaction interface between the EhPC4 and EhCFIm25 structures was analyzed using LigPlot+ software (v 2.3.1). Forty-four residues within the N-terminal region of EhPC4 were identified as interacting with EhCFIm25 (Figure 6A). Conversely, thirty-nine residues of EhCFIm25 were found to mediate the interaction with EhPC4. Residues M252 and Y254 of EhCFIm25 are likely particularly important, as they are conserved across all five interaction complexes (C1–C5) (Figure 6B). Based on this, the conserved interactions common to all complexes were analyzed. Three specific interactions were conserved in three of the five interaction complexes: EhPC4(D133)–EhCFIm25(Y254), EhPC4(I143)–EhCFIm25(G255), and EhPC4(K151)–EhCFIm25(R249) (Figure 6C).

**Figure 7:**
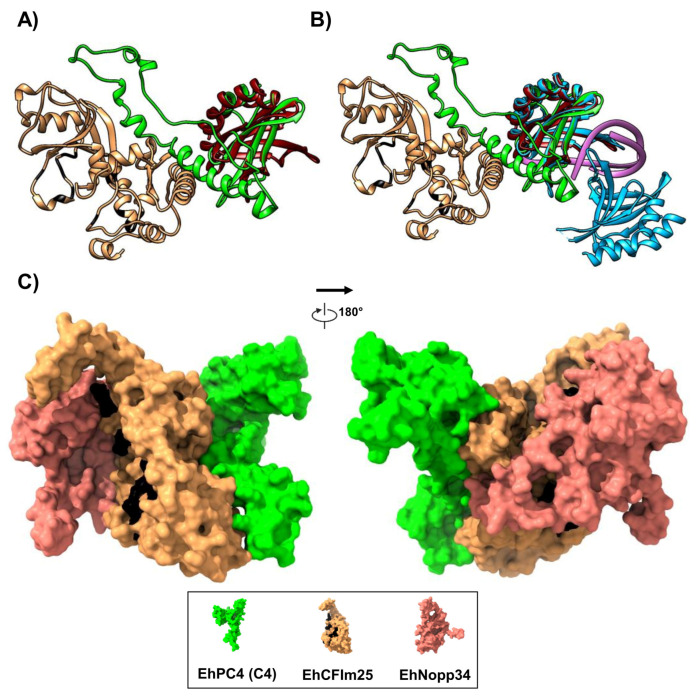
Comparison of the molecular complexes formed by EhPC4 (C4)-EhCFIm25. (**A**) Interaction of EhPC4-EhCFIm25 (C4) model with human PC4 dimer (1PCF, dark red). (**B**) Interaction of EhPC4-EhCFIm25 (C4) model with human PC4 dimer and PC4 binding domain-DNA, marked in blue and purple, respectively (6YCS). (**C**) EhPC4-EhCFIm25 (C4) model with NOPP34. Amino acids of EhCFIm25 that bind to RNA (GUUG motif) are indicated in black.

Finally, the C4 complex of EhCFIm25 and EhPC4 was superimposed with the C-terminal domain of human PC4 (PDB: 1PCF) and with the C-terminal domain of the human PC4 dimer bound to DNA (PDB: 6YCS). This analysis revealed that the EhPC4 domains responsible for dimerization and DNA binding remain accessible in the selected model (Figure 7A,B). Furthermore, the EhPC4(C4)-EhCFIm25 complex was superimposed with the previously reported molecular docking model of EhCFIm25 bound to EhNopp34 [3]. This comparison indicated that the EhPC4(C4) model does not occlude the binding interface of EhCFIm25 for other proteins involved in pre-mRNA polyadenylation in *E. histolytica*, as each protein interacts via a distinct recognition region. It is also important to note that the RNA-binding sites of EhCFIm25 remain accessible in the analyzed complexes. These findings suggest a plausible interaction among these three proteins in a biological context, thereby supporting a functional link between transcription and mRNA polyadenylation processes in *E. histolytica* (Figure 7C).

## 4. Discussion

The growing evidence of crosstalk between transcription, splicing, and polyadenylation machinery suggests that mRNA biogenesis is a continuum of highly integrated events rather than a series of independent steps. In this study, we used Far-Western blotting assays to demonstrate a physical interaction between the polyadenylation factor EhCFIm25 and the transcription factor EhPC4 in the protozoan parasite *E. histolytica*. While the specificity of the in vitro interaction was supported by the absence of signal in the antibody-only controls, it is further reinforced by previous studies using the same recombinant protein platform. The ability of EhCFIm25 to specifically bind EhPAP while other components like Ehstf64 fail to do so under identical conditions [9] demonstrates the selectivity of our Far-Western blot system. These precedents, combined with the structural consistency observed in our in silico docking models, suggest that the EhPC4–EhCFIm25 association is a specific biochemical event rather than a technical artifact.

These findings provide experimental support for the computational PPI models, which position EhCFIm25 as a critical bottleneck protein [3]. The ability of EhCFIm25 to interact with both the catalytic hub (EhPAP) [9] and a transcriptional coactivator (EhPC4) suggests that it may serve as a scaffold or “molecular bridge.” This bridge could facilitate the early recruitment of the polyadenylation machinery to the transcription site, ensuring efficient processing as the nascent RNA emerges from the RNA Polymerase II. In yeast that lacks the homolog of CFIm25, Sub1 (the homolog of PC4) is responsible for the tight connection between transcription and mRNA processing. Following transcription initiation, PC4/Sub1 dissociates from transcriptional activators and general transcription factors. It becomes available to bind another polyadenylation factor, namely Rna15p (the homolog of CstF64), to avoid premature transcription termination [13].

This interaction is particularly relevant in *E. histolytica*, where rapid environmental adaptation is key to pathogenesis. Therefore, disrupting this link between EhPC4 and EhCFIm25 would be an interesting strategy for parasite control. The link between EhPC4, a regulator of migration and destruction of host epithelium [14,15], and EhCFIm25, essential for viability [6,7,8], suggests a coordinated regulatory axis that could synchronize the transcriptional response with the 3′ end mRNA processing required for stable protein synthesis. In human, RNA polymerase II transcription is tightly coupled to pre-mRNA processing, with capping, splicing, and 3′ end cleavage/polyadenylation occurring largely co transcriptionally on the nascent transcript [24].

The 500 ns MD trajectory provides a robust sampling of the conformational landscape of EhPC4. The significant displacement of residue S54 (up to 52 Å) in Cluster C4 highlights the intrinsic disorder of the N-terminal domain, a feature that facilitates the ‘search-and-capture’ mechanism to recruit EhCFIm25. From a computational perspective, the stability of the RMSD and Rg values across independent replicates ensures that the docking models are based on biologically relevant conformers rather than transient states. Human PC4 (Sub1 in yeast) contains a highly flexible, intrinsically disordered N terminal domain that modulates protein–protein interactions of the PC4 core, while PC4 directly interacts with p53 to regulate p53 function [25,26]. Therefore, the results of the docking analysis could suggest that the N-terminal domain of EhPC4 observed in the C4 cluster structure could regulate protein binding during transcription and mRNA processing through a structural mechanism. However, the possibility that the models of the other clusters (C1, C2, C3 and C5) may have functional relevance for the regulation of these processes should not be ruled out, and this should be explored in greater depth in future studies.

Our contact map analysis (Figure 6) demonstrates a high-occupancy interaction zone at the C-terminus of EhCFIm25. The persistence of residues M252 and Y254 across all docking clusters underscores their role as critical anchoring points. In human CFIm, the integrity of the CFIm25 (NUDT21) C terminal Nudix fold is required to form the CFIm25 homodimer that provides the structural scaffold for assembly of the heterotetrametric CFIm complex with CFIm68/59 [27,28,29]. Importantly, the structural superposition with PDB: 6YCS confirms that the EhPC4-EhCFIm25 assembly preserves the spatial orientation required for DNA binding. This lack of steric hindrance suggests a co-transcriptional model where EhPC4 bridges the genomic DNA and the polyadenylation machinery, an architectural arrangement that optimizes the mRNA maturation rate. In human, PC4 is a DNA-binding coactivator that functions within promoter-bound multi-protein assemblies, cooperating with activators and components of the basal transcription machinery during TFIIA–TFIID–promoter complex formation and requiring TFIIH/TAFs for productive activation [30,31].

The integration of Far-Western validation with MDs and protein docking reveals a specialized molecular bridge in *E. histolytica*. Unlike higher eukaryotes, where these complexes are highly transient, the thermodynamic stability (∆G < −8.7 kcal/mol) of the EhPC4-EhCFIm25 complex suggests a constitutive coupling mechanism, which is in the range commonly reported for reversible, regulatory protein–protein interactions [32,33]. Given that pre-mRNA processing machinery is essential and pharmacologically vulnerable in protozoa (e.g., CPSF3/73 in apicomplexans), and that EhCFIm25 is required for survival/virulence in *E. histolytica*, the EhPC4–EhCFIm25 interface constitutes a plausible target for the rational design of inhibitors that disrupt the functional coupling between the transcriptional response and 3′ mRNA processing [34,35,36]. To date, amoebiasis remains a major public health problem in many developing regions; there is no vaccine and the main treatment relies on metronidazole that was developed in the 1960s and has several side effects that limit its use. Therefore, the identification of a molecule able to affect the EhPC4–EhCFIm25 interface would represent a valuable therapeutic strategy that certainly would have a huge impact for the control of this human parasite disease.

## 5. Conclusions

In this work, we provided a comprehensive structural and functional characterization of the interaction between the transcription factor EhPC4 and the polyadenylation factor EhCFIm25 in *Entamoeba histolytica*. By integrating 500 ns molecular dynamics simulations with molecular docking and Far-Western validation, we demonstrated that this interaction is not only thermodynamically stable but also structurally non-competitive. The identification of a high-occupancy ‘hotspot’ at the C-terminal region of EhCFIm25 (residues 249–255) reveals a specialized interface that facilitates the recruitment of transcriptional machinery without occluding DNA or RNA binding domains. This architectural arrangement supports a co-transcriptional coupling model, where EhCFIm25 acts as a central molecular scaffold bridging early synthesis and late-stage mRNA processing. Our findings underscore the complexity of the parasite’s interactome and identify the EhPC4-EhCFIm25 interface as a novel, plausible target for disrupting the regulatory axis essential for the virulence and survival of this human pathogen.

## Data Availability

The original contributions presented in this study are included in the article. Further inquiries can be directed to the corresponding author.

## Abbreviations

The following abbreviations are used in this manuscript:

CFIm	Cleavage factor I
DAB	Diaminobenzidine
IPTG	isopropyl β-D-thiogalactopyranoside
MD	Molecular dynamics
PC4	Positive Coactivator 4
PME	Particle Mesh Ewald
PPI	Protein–protein interaction
Rg	Radius of gyration
RMSD	Root mean square deviation
RMSF	Root mean-square-fluctuation
ssDNA	single-stranded DNA
UTR	Untranslated region
